# Time to diagnostic resolution after an uncertain screening mammogram in an underserved population

**DOI:** 10.1002/cam4.2970

**Published:** 2020-03-11

**Authors:** Anita J. Kumar, Darcy Banco, Elise E. Steinberger, Joanna Chen, RuthAnn Weidner, Shital Makim, Susan K. Parsons

**Affiliations:** ^1^ Institute for Clinical Research and Health Policy Studies Tufts Medical Center Boston MA USA; ^2^ Department of Medicine Tufts University School of Medicine Boston MA USA; ^3^ Department of Radiology Tufts Medical Center Boston MA USA

**Keywords:** diagnostic resolution, uncertain screening mammogram

## Abstract

**Background:**

Screening mammography has reduced breast cancer–associated mortality worldwide. Approximately 10% of patients require further diagnostic testing after an uncertain screening mammogram (Breast imaging reporting and data system [BI‐RADS] = 0), and time to diagnostic resolution varies after BI‐RADS = 0 screening mammogram. There is little data about factors associated with diagnostic resolution in patients of Chinese origin (“Chinese”) receiving care in the US.

**Methods:**

We performed a retrospective analysis to identify patterns of diagnostic resolution in an urban US hospital with a large population of Chinese patients. We evaluated whether location of primary care provider (PCP) impacted time to resolution among Chinese patients, hypothesizing that patients with a PCP outside of the hospital would have longer time to diagnostic resolution than those patients with a PCP within the institution.

**Results:**

Between 2015 and 2016, 368 patients at Tufts Medical Center (Tufts MC) had resulting BI‐RADS = 0 after screening mammogram. The majority of patients (341/368, 93%) achieved diagnostic resolution with median time to resolution 27 days (Q1: 14, Q3: 40). Seven percent (27/368) never achieved resolution. Among those with diagnostic resolution, 10% of patients required >60 days to achieve resolution. Chinese origin, no previous breast cancer, subsidized insurance, and outside referring physician were associated with longer time to resolution in univariable analysis. In multivariable regression, after adjusting for age, insurance, marital status, and prior breast cancer, Chinese patients with Tufts MC PCP experienced timelier diagnostic resolution vs Chinese patients without a Tufts MC PCP (hazard ratio [HR] = 1.85, *P* = .02). Location of PCP did not impact time to resolution among non‐Chinese patients.

**Conclusion:**

We identified patterns of diagnostic resolution in an urban hospital with a large historically underserved population. We found that Chinese patients without integrated primary care within the institution are at risk for delayed diagnostic resolution. Future interventions need to target at‐risk patients to prevent loss of follow‐up after uncertain screening mammogram.

## Graphical abstract

In an urban hospital, >90% of patients achieve diagnostic resolution after uncertain screening mammogram. Chinese patients without an integrated primary care provider (PCP) are at risk of less timely diagnostic resolution than Chinese patients with integrated PCP.

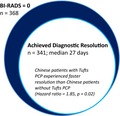

## 1 INTRODUCTION

While the introduction of screening mammography in breast cancer detection has markedly reduced breast cancer–associated mortality worldwide, there remain differences in mortality among non‐White patients compared with White patients.^1^ This disparity may be in part due to biology,^2^, ^3^, ^4^ but also may be due to delays in diagnosis and treatment after abnormal screening mammogram.^1^, ^3^, ^5^, ^6^, ^7^, ^8^ Approximately 10% of women who undergo screening mammography require further imaging and/or biopsy to achieve diagnostic resolution—that is, a “final” verdict whether the lesion is cancer.^9^ The interpretation of screening mammograms is defined by the breast imaging reporting and data system (BI‐RADS), which relates mammographic findings to the likelihood of breast cancer.^10^ Breast imaging reporting and data system reports results on a scale from 0 to 6, with BI‐RADS = 1‐3 representing negative, benign, and probably benign findings. BI‐RADS = 4‐6 results indicate findings suspicious, suggestive, or proven of malignancy. Finally, BI‐RADS = 0 indicates a possible finding that needs additional workup, necessitating further testing to achieve diagnostic resolution, hereafter referred to as “uncertain screening mammogram.”

Prior studies have demonstrated that, after controlling for race and a patient's demographic characteristics, there may be wide variations in the time to diagnostic resolution among health centers, suggesting that the method of care delivery may be a more proximal determinant of timely care.^11^ Furthermore, patient barriers such as language, confusion regarding follow‐up testing, transportation to appointments, and fear of results of further testing have also been explored as possible limitations to timely diagnostic resolution.^12^, ^13^ While there exists substantial data about minority patients experiencing longer times to diagnostic resolution, there is a paucity of data on historically underserved populations, such as patients of Chinese origin (“Chinese”).

We performed a single‐center retrospective study to identify what proportion of patients achieved diagnostic resolution and in what time frame after uncertain screening mammogram in an urban hospital with a large Chinese population. We chose to examine those with BI‐RADS = 0 mammograms, as these require follow‐up to achieve resolution. We also sought to identify whether closer integration of care with a primary care provider (PCP) influenced time to resolution among Chinese and non‐Chinese patients.

## 2 METHODS

### 2.1 Study population

Tufts Medical Center (Tufts MC) is a 415‐bed tertiary care hospital located in the Chinatown neighborhood of Boston, MA. Tufts Medical Center serves patients from the Chinatown area of Boston, as well as patients located in surrounding neighborhoods within Greater Boston. We included patients who underwent screening mammography at Tufts MC with resulting BI‐RADS = 0 classification between 1 October 2015 and 30 September 2016, to allow for a minimum follow‐up period of 1 year for all patients. Patients were excluded if they died prior to the end of the study period without achieving diagnostic resolution.

### 2.2 Outcomes and data abstraction

The primary outcome of this study was to evaluate what proportion of patients achieved diagnostic resolution after a screening mammogram with uncertain result (BI‐RADS = 0). The secondary outcome was to identify whether Chinese patients experienced a longer time to achieve diagnostic resolution than other groups, and, whether having a Tufts MC PCP may influence this time. Diagnostic resolution was defined as a definitive diagnostic test, characterized as BI‐RADS = 1, 2, or 3 imaging mammogram or ultrasound, or a definitive biopsy. We identified patients through the Tufts MC Radiology mammography database, which tracks all patients who underwent screening mammography at Tufts MC. Trained study staff abstracted study data from the hospital's electronic medical records and input it into the Research Electronic Data Capture hosted at the Tufts Clinical and Translational Science Institute.^14^, ^15^ Research Electronic Data Capture is a secure, web‐based software platform designed to support data capture for research studies, providing (a) an intuitive interface for validated data capture; (b) audit trails for tracking data manipulation and export procedures; (c) automated export procedures for seamless data downloads to common statistical packages; and (d) procedures for data integration and interoperability with external sources.

We collected data on patient demographic characteristics (age, address, insurance plan, race/ethnicity, primary language, and history of cancer), referring provider location, and postreferral testing. We used insurance as a surrogate for poverty rather than poverty level based on zip code. This was done to avoid confounding by gentrification of city neighborhoods. Insurance categories included private only (including Medicare patients who carried private gap insurance) or subsidized. For patients with more than 1 screening mammogram during the study period, only the first mammogram was included in the primary dataset. This study was approved by the Tufts MC Institutional Review Board.

### 2.3 Statistical analysis

Baseline demographic, clinical, and provider characteristics were described using medians and description of 25th percentile (Q1) and 75th percentile (Q3), or frequencies (percentages). One patient who achieved diagnostic resolution on the same day as her screening mammogram was assumed to have time to diagnostic resolution of 1 day. We compared time with resolution among key demographics using the Wilcoxon rank‐sum test. Chi‐squared tests were used to compare categorical variables. We performed multivariable Cox regression analysis to identify whether Chinese patients (defined as Chinese race or primary language a Chinese dialect) were slower to achieve diagnostic resolution, after adjusting for other sociodemographic factors. Based on a priori hypothesis that Chinese patients with PCPs within Tufts MC would be more likely to achieve diagnostic resolution more quickly, we tested for an interaction between Chinese patients and location of PCP (within Tufts MC vs outside of Tufts MC). A hazard ratio of >1 indicates a faster time to achieving resolution, whereas a hazard ratio < 1 indicates a slower time to achieve resolution. We tested for proportional hazard ratio assumptions using Schoenfeld residuals. Statistical tests were two sided with an *α* of 0.05 and analyses were conducted using Stata Version 15.1.

## 3 RESULTS

### 3.1 Patient characteristics

We identified 368 patients who underwent screening mammogram at Tufts MC with BI‐RADS = 0 result during the study period. The median age of study patients was 57 years (Q1 = 49, Q3 = 65). Over half (53%) of patients were Caucasian and 84/368 (22.8%) of patients identified as Asian. Among Asians, the majority of patients (83.3%, 70/84) identified as Chinese. Among all patients, 57/368 (15.5%) identified their primary language as a Chinese dialect. While most patients used private insurance for their medical care (74.2%), the remainder received subsidized insurance.

Majority of patients (86.7%) were undergoing routine screening mammography. Ten percent of patients had a previously documented breast cancer. A small percentage (1%) was documented to have high‐risk status (family history and prior radiation) or other reasons for mammography (eg, dense breasts, pain, fibrocystic disease).

Most patients were referred for their screening mammogram by a provider at Tufts MC (85.6%), and 77.1% of Tufts MC referrals originated from primary care. We also noted a limited number of referrals from community health centers, private practices, and PCPs outside of Tufts MC (Table 1).

**TABLE 1:** Patient characteristics

Characteristic	Total (%) n = 368
Age (median) (Q1, Q3)	57 (49, 65)
Race/ethnicity	
White/Caucasian	195 (53)
Asian	84 (22.9)
Black	52 (14.1)
Hispanic/Latino	17 (4.6)
Unknown or other	20 (5.4)
Primary language	
English	286 (77.7)
Chinese (Mandarin or Cantonese)	57 (15.5)
Spanish	12 (3.3)
Other	13 (3.5)
Requires interpreter	57 (15.5%)
Marital status	
Single	101 (27.4)
Currently married/partnered	224 (60.9)
Widowed, divorced, separated	40 (10.9)
Unknown or other	3 (0.8)
Insurance	
Private only	273 (74.2)
Any subsidized	95 (25.8)
Medicare ± MassHealth	32 (33.7)
MassHealth alone or secondary (no Medicare)	56 (58.9)
Exchange	1 (1.1)
Health Safety Net	6 (6.3)
Reason for screening	
Routine screening	319 (86.7)
Previous breast cancer	40 (10.9)
High‐risk status features	4 (1.1)
Other	5 (1.3)
Referring provider location/type	
Hospital	318 (86.4)
Tufts Medical Center	315 (99)
PCP	243 (77)
Subspecialist	72 (23)
Outside hospital	3 (1)
Community health center	7 (1.9)
PCP	2 (28.6)
Subspecialist	5 (71.4)
Community‐based private practice	41 (11.2)
PCP	37 (90.2)
Subspecialist	4 (9.8)
Other PCP	2 (0.5)

Abbreviation: PCP, primary care provider.

#### 3.1.1 Time to diagnostic resolution

Among 368 patients evaluable for time to resolution, 341/368 (93%) achieved diagnostic resolution, of which 90% resolved within 60 or fewer days (median time 27 days; Q1 = 14, Q3 = 40). Of note, 27/368 (7%) did not resolve with a minimum follow‐up of 1 year after screening mammogram.

Of 363 patients with complete biopsy data, 51/363 (14%) underwent biopsy prior to diagnostic resolution. Median time from screening mammogram to biopsy was 34 days (Q1 = 20, Q3 = 53). Biopsy yielded documentation of new diagnosis of breast cancer in 20/51 biopsied cases (39.2%).

Among the 341 patients who achieved resolution, 23 (6.7%) were diagnosed with breast cancer. Time to diagnostic resolution did not differ among those diagnosed with cancer (median 22 days; Q1 = 11, Q3 = 51) vs those who resolved without cancer (median 27 days; Q1 = 14, Q3 = 39), *P* = .91. Of those patients diagnosed with cancer, 21/23 were documented to have met with a breast oncologist at a median of 11 days (Q1 = 8, Q3 = 13) after diagnosis.

Median time to resolution was longer in Chinese (34 days vs 24 days for non‐Chinese patients, *P* < .01), patients without a history of breast cancer (28 days vs 18 days in patients with prior breast cancer, *P* < .01), subsidized insurance (31 days vs 25 days for privately insured patients, *P* = .03), and location of referring physician (non‐Tufts MC physician 32 days vs Tufts MC physicians 26 days, *P* = .04).

For patients who did not achieve diagnostic resolution, we found no difference in most patient demographics including spoken language (Chinese vs other, *P* = .52), subsidized vs private insurance (*P* = .35), reason for screening (*P* = .09), need for an interpreter (*P* = .74), and ordering site (Tufts MC vs outside of Tufts MC, *P* = .53). We found that married or partnered patients were more likely to achieve resolution than nonpartnered patients (single, widowed, and divorced; 95% vs 89%, *P* = .02).

#### 3.1.2 Univariable regression

In univariable analysis we found that while Chinese patients experienced longer time to diagnostic resolution (HR 0.75, 95% CI 0.57‐0.97, *P* = .03), they did not have different odds of achieving diagnostic resolution compared with non‐Chinese patients (odds ratio = 1.43, 95% CI 0.48‐4.3, *P* = .52). Major differences between Chinese patients and non‐Chinese patients in our cohort included the proportion of people with subsidized insurance (45.8% vs 21%, *P* < .01), the proportion of people with history of breast cancer (4.2% vs 12.5%, *P* = .04), the proportion of patients married (22.9% vs 43.3%, *P* < .01), and the proportion of people with a PCP outside of Tufts MC (40.3% vs 22.3%, *P* < .01). Age did not differ between Chinese vs non‐Chinese patients (median 56 years vs 59 years, *P* = .23).

#### 3.1.3 Multivariable logistic regression

In multivariable analysis, after adjusting for age, insurance, marital status, and previous breast cancer, we identified an interaction between Chinese patients and PCP location that impacted time to diagnostic resolution (interaction *P* = .01). Chinese patients with a Tufts MC PCP achieved timelier diagnostic resolution than Chinese patients without a Tufts MC PCP (HR = 1.85, 95% CI 1.12‐3.05, *P* = .02). In contrast, having a Tufts MC PCP did not significantly impact time to diagnostic resolution among non‐Chinese patients (HR = 0.86 95% CI: 0.64‐1.16, *P* = .33) (Table 2). We also found a similar association among all primary English vs non‐English speaking patients: non‐English speaking patients with Tufts MC PCP were faster to achieve resolution than non‐English speaking patients without a Tufts MC PCP (HR = 1.89, 95% CI 1.16‐3.06, *P* = .01), interaction *P* < .01. English speaking patients experienced no difference in time to resolution based on PCP (HR = 0.86, 95% CI = 0.64‐1.16, *P* = .34). We did not find a significant interaction among patients requiring interpreter and presence of Tufts MC‐based PCP on time to diagnostic resolution (*P* = .23).

**TABLE 2:** Impact of Chinese ethnicity and PCP on time to diagnostic resolution

Variable (reference)	Univariable regression		Multivariable regression (n = 338)		
	HR	*P* value	HR	95% CI	*P* value
Effect of Tufts MC PCP*					
Chinese patients (ref: no Tufts PCP)	1.78	.02	1.85	1.12‐3.06	.02
Non‐Chinese patients (ref: no Tufts PCP)	0.76	.07	0.86	0.64‐1.16	.33
Age, by year	1.00	.83	1.00	0.99‐1.01	.75
Subsidized Insurance (ref: Private)	0.80	.07	0.94	0.72‐1.24	.68
Previous breast cancer (ref: no previous breast cancer)	1.64	<.01	1.58	1.11‐2.26	.01
Married/Partnered (ref: single/widowed/divorced)	1.26	.04	1.28	0.99‐1.64	.06

Abbreviation: PCP, primary care provider.

* Interaction *P* = .01.

## 4 DISCUSSION

We present a comprehensive analysis of time to diagnostic resolution after an uncertain screening mammogram in a tertiary care hospital that provides care for a significant proportion of the Chinese population within Boston, MA. In our study, over 90% of patients achieved diagnostic resolution with median time to resolution of 27 days. This finding is consistent with prior studies in minority patients have cited follow‐up rates in similar ranges.^16^, ^17^, ^18^, ^19^ We also found that, consistent with prior literature, 90% of patients who achieved diagnostic resolution do so within 60 days.^16^ The landmark of 60 days has been established through quality measures by the National Breast and Cervical Cancer Early Detection Program, a program sponsored by the Centers for Disease Control & Prevention that provides screening services to underserved woman.^19^ Yet, while most patients achieved diagnostic resolution within 60 days, biopsies on average took place >30 days after an uncertain screening mammogram, which is longer than previously published data.^20^ This may have been due to delay due to patient factors, scheduling challenges, or need for further imaging prior to biopsy.

Interestingly, demographics of patients who achieved diagnostic resolution were similar to those who did not achieve diagnostic resolution, with the exception of marital status, as partnered patients were more likely to achieve diagnostic resolution. Lack of differences among these 2 groups may partly reflect the small number of patients in our study who did not achieve diagnostic resolution. Similarly, we did not investigate the impact of lack of housing, employment, transportation, and other such potential social determinants that may impact follow‐up time.^17^ Nonetheless, our findings highlight that marital and social support may significantly impact ability to follow‐up after uncertain mammogram.

Among the patients who received a new diagnosis of breast cancer, 75% of newly diagnosed patients met with a breast oncologist within 2 weeks, with the longest wait from diagnosis to oncology appointment being 21 days. Researchers have found that length of time between cancer diagnosis and treatment initiation may affect survival in certain settings, particularly for patients with early‐stage breast cancer, lung cancer, and pancreatic cancer. Studies have shown that factors contributing to time to treatment initiation include changes in facilities and treatment at an academic center. 21 Khorana et al described a multidisciplinary program that reduced time to initiation by one‐third in a large academic medical center. The study highlights the need for a team approach in the setting of accounting for patient preferences when addressing reductions in delays to treatment initiation.^22^

These approaches may extend to the most striking finding in our analysis: the critical relationship between Chinese ethnicity and PCP location on time to diagnostic resolution. Chinese patients with a Tufts MC PCP were timelier in achieving resolution compared with Chinese patients without a Tufts MC PCP, after adjusting for socioeconomic status. This finding was consistent when looking at all patients who did not speak English as their primary language (majority were Chinese speaking), suggesting that the impact of language barriers may be worsened by not having a PCP that is closely integrated with the breast health center.

How does this impact cancer care delivery? One potential solution is to extend the existing patient navigation program to this phase of care. Patient navigation has been well recognized as a method to improve follow‐up for care in at‐risk populations, including minority patients.^18^, ^20^, ^23^ Currently, the institution's patient navigation program, focusing on patients of Chinese origin and/or lower socioeconomic status, is offered to patients following the establishment of the cancer diagnosis. The results of this study suggest that for vulnerable patients, such as those without a Tufts MC PCP, patient navigation services may be warranted even before a cancer diagnosis as patients undergo additional diagnostic workup. Another solution is to identify at‐risk populations, triaging such patients for urgent follow‐up or even same‐day diagnostic testing prior to leaving the hospital after screening mammogram to improve this healthcare disparity. Additionally, navigators may serve as a bridge between the referral site and referring PCP, when outside of the referral site. This may be helpful in reducing communication challenges between the PCP and breast health center. Lastly, understanding cultural barriers, such as understanding of their results,^24^ fear of follow‐up testing, or beliefs about additional testing, must be addressed to ensure timely follow‐up after an uncertain screening mammogram.

We acknowledge limitations of our study. With a retrospective single‐center study, our findings may not be generalizable to a larger population given our catchment population. Also, we note that with a minimum follow‐up period of 1 year, 27/368 patients did not achieve resolution, and we do not have data for reasons why these patients were lost to follow‐up. However, our single‐center analysis allowed us to capture demographic and variables about PCP with minimal missing data. We believe this is a strength of this study. Lastly, we did not investigate subcategories within private insurance, including deductibles. Patients enrolled in high‐deductible healthcare plans may experience delays in diagnosis and treatment, which may identify additional at‐risk populations among those with private insurance.^25^

In conclusion, we present an analysis of times to diagnostic resolution in a hospital that serves a sizeable proportion of Chinese patients. We find that, while most patients achieve diagnostic resolution within 60 days, patients without a PCP within the hospital experience longer times to diagnostic resolution—even after adjusting for other sociodemographic factors. Future interventions are needed to reduce disparities in diagnostic testing among Chinese patients after an uncertain screening mammogram.

## CONFLICTS OF INTEREST

None declared.

## AUTHOR CONTRIBUTION

A.J.K.: data analysis; methodology; writing‐original draft, manuscript review, and editing. R.A.W.: database development; data interpretation; manuscript preparation—review and editing. D.B., E.S., J.C.: data collection and analysis, manuscript preparation—review and editing. S.M.: conceptualization, data interpretation, manuscript preparation—review and editing. S.K.P: conceptualization; formal analysis; funding acquisition; methodology; data interpretation, manuscript preparation—review and editing.

## Data Availability

The author elects to not share data.
